# Implications of mappings between International Classification of Diseases clinical diagnosis codes and Human Phenotype Ontology terms

**DOI:** 10.1093/jamiaopen/ooae118

**Published:** 2024-11-18

**Authors:** Amelia L M Tan, Rafael S Gonçalves, William Yuan, Gabriel A Brat, Robert Gentleman, Isaac S Kohane, The Consortium for Clinical Characterization of COVID-19 by EHR (4CE), Aaron J Masino, Adeline Makoudjou, Adem Albayrak, Alba Gutiérrez-Sacristán, Alberto Zambelli, Alberto Malovini, Aldo Carmona, Alexander Hoffmann, Alexandre Gramfort, Alon Geva, Alvar Blanco-Martínez, Amelia L M Tan, Ana I Terriza-Torres, Anastasia Spiridou, Andrea Prunotto, Andrew M South, Andrew K Vallejos, Andrew Atz, Anita Burgun, Anna Alloni, Anna Maria Cattelan, Anne Sophie Jannot, Antoine Neuraz, Antonio Bellasi, Anupama Maram, Arianna Dagliati, Arnaud Sandrin, Arnaud Serret-Larmande, Arthur Mensch, Ashley C Pfaff, Ashley Batugo, Ashok K Krishnamurthy, Atif Adam, Audrey Dionne, Batsal Devkota, Bertrand Moal, Bing He, Brendin R Beaulieu-Jones, Brett K Beaulieu-Jones, Brian D Ostasiewski, Bruce J Aronow, Bryce W Q Tan, Byorn W L Tan, Carlo Torti, Carlos Sáez, Carlos Tadeu Breda Neto, Charles Sonday, Charlotte Caucheteux, Chengsheng Mao, Chiara Zucco, Christel Daniel, Christian Haverkamp, Chuan Hong, Clara-Lea Bonzel, Cinta Moraleda, Damien Leprovost, Daniel A Key, Daniela Zöller, Danielle Pillion, Danielle L Mowery, Danilo F Amendola, Darren W Henderson, David A Hanauer, Deanne M Taylor, Demian Wassermann, Derek Y Hazard, Detlef Kraska, Diego R Mazzotti, Domenick Silvio, Douglas S Bell, Douglas A Murad, Elisa Salamanca, Emily Bucholz, Emily J Getzen, Emily R Pfaff, Emily R Schriver, Emma M S Toh, Enea Parimbelli, Enrico M Trecarichi, Fatima Ashraf, Fernando J Sanz Vidorreta, Florence T Bourgeois, Francesca Sperotto, François Angoulvant, Gabriel A Brat, Gael Varoquaux, Gilbert S Omenn, Giuseppe Agapito, Giuseppe Albi, Griffin M Weber, Guillaume Verdy, Guillaume Lemaitre, Gustavo Roig-Domínguez, Hans U Prokosch, Harrison G Zhang, Hossein Estiri, Ian D Krantz, Isaac S Kohane, Jacqueline P Honerlaw, Jaime Cruz-Rojo, James B Norman, James Balshi, James J Cimino, James R Aaron, Janaina C C Santos, Jane W Newburger, Janet J Zahner, Jason H Moore, Jayson S Marwaha, Jean B Craig, Jeffrey G Klann, Jeffrey S Morris, Jihad Obeid, Jill-Jênn Vie, Jin Chen, Jiyeon Son, Joany M Zachariasse, John Booth, John H Holmes, José Luis Bernal-Sobrino, Juan Luis Cruz-Bermúdez, Judith Leblanc, Juergen Schuettler, Julien Dubiel, Julien Champ, Karen L Olson, Karyn L Moshal, Kate F Kernan, Katie Kirchoff, Kavishwar B Wagholikar, Kee Yuan Ngiam, Kelly Cho, Kenneth D Mandl, Kenneth M Huling, Krista Y Chen, Kristine E Lynch, L Nelson Sanchez-Pinto, Lana X Garmire, Larry Han, Lav P Patel, Lemuel R Waitman, Leslie Lenert, Li L L J Anthony, Loic Esteve, Lorenzo Chiudinelli, Luca Chiovato, Luigia Scudeller, Malarkodi Jebathilagam Samayamuthu, Marcelo R Martins, Marcos F Minicucci, Maria Clara Saad Menezes, Margaret E Vella, Maria Mazzitelli, Maria Savino, Marianna Milano, Marina P Okoshi, Mario Cannataro, Mario Alessiani, Mark S Keller, Martin Hilka, Martin Wolkewitz, Martin Boeker, Maryna Raskin, Mauro Bucalo, Meghan R Hutch, Mélodie Bernaux, Michele Beraghi, Michele Morris, Michele Vitacca, Miguel Pedrera-Jiménez, Mohamad Daniar, Mohsin A Shah, Molei Liu, Monika Maripuri, Mundeep K Kainth, Nadir Yehya, Nandhini Santhanam, Nathan P Palmer, Ne Hooi Will Loh, Neil J Sebire, Nekane Romero-Garcia, Nicholas W Brown, Nicolas Paris, Nicolas Griffon, Nils Gehlenborg, Nina Orlova, Noelia García-Barrio, Olivier Grisel, Pablo Rojo, Pablo Serrano-Balazote, Paolo Sacchi, Patric Tippmann, Patricia Martel, Patricia Serre, Paul Avillach, Paula S Azevedo, Paula Rubio-Mayo, Petra Schubert, Pietro H Guzzi, Piotr Sliz, Priyam Das, Qi Long, Rachel B Ramoni, Rachel S J Goh, Rafael Badenes, Raffaele Bruno, Ramakanth Kavuluru, Riccardo Bellazzi, Richard W Issitt, Robert W Follett, Robert L Bradford, Robson A Prudente, Romain Bey, Romain Griffier, Rui Duan, Sadiqa Mahmood, Sajad Mousavi, Sara Lozano-Zahonero, Sara Pizzimenti, Sarah E Maidlow, Scott Wong, Scott L DuVall, Sébastien Cossin, Sehi L'Yi, Shawn N Murphy, Shirley Fan, Shyam Visweswaran, Siegbert Rieg, Silvano Bosari, Simran Makwana, Stéphane Bréant, Surbhi Bhatnagar, Suzana E Tanni, Sylvie Cormont, Taha Mohseni Ahooyi, Tanu Priya, Thomas P Naughton, Thomas Ganslandt, Tiago K Colicchio, Tianxi Cai, Tobias Gradinger, Tomás González González, Valentina Zuccaro, Valentina Tibollo, Vianney Jouhet, Víctor Quirós-González, Vidul Ayakulangara Panickan, Vincent Benoit, Wanjiku F M Njoroge, William A Bryant, William Yuan, Xin Xiong, Xuan Wang, Ye Ye, Yuan Luo, Yuk-Lam Ho, Zachary H Strasser, Zahra Shakeri Hossein Abad, Zongqi Xia, Kernan F Kate, Alejandro Hernández-Arango, Eli L Schwamm

**Affiliations:** Department of Biomedical Informatics, Harvard Medical School, Boston, MA 02115, United States; Center for Computational Biomedicine, Harvard Medical School, Boston, MA 02115, United States; Department of Biomedical Informatics, Harvard Medical School, Boston, MA 02115, United States; Department of Biomedical Informatics, Harvard Medical School, Boston, MA 02115, United States; Center for Computational Biomedicine, Harvard Medical School, Boston, MA 02115, United States; Department of Biomedical Informatics, Harvard Medical School, Boston, MA 02115, United States

**Keywords:** ontology, data interoperability, ontology interoperability

## Abstract

**Objective:**

Integrating electronic health record (EHR) data with other resources is essential in rare disease research due to low disease prevalence. Such integration is dependent on the alignment of ontologies used for data annotation. The international classification of diseases (ICD) is used to annotate clinical diagnoses, while the human phenotype ontology (HPO) is used to annotate phenotypes. Although these ontologies overlap in the biomedical entities they describe, the extent to which they are interoperable is unknown. We investigate how well aligned these ontologies are and whether such alignments facilitate EHR data integration.

**Materials and Methods:**

We conducted an empirical analysis of the coverage of mappings between ICD and HPO. We interpret this mapping coverage as a proxy for how easily clinical data can be integrated with research ontologies such as HPO. We quantify how exhaustively ICD codes are mapped to HPO by analyzing mappings in the unified medical language system (UMLS) Metathesaurus. We analyze the proportion of ICD codes mapped to HPO within a real-world EHR dataset.

**Results and Discussion:**

Our analysis revealed that only 2.2% of ICD codes have direct mappings to HPO in UMLS. Within our EHR dataset, less than 50% of ICD codes have mappings to HPO terms. ICD codes that are used frequently in EHR data tend to have mappings to HPO; ICD codes that represent rarer medical conditions are seldom mapped.

**Conclusion:**

We find that interoperability between ICD and HPO via UMLS is limited. While other mapping sources could be incorporated, there are no established conventions for what resources should be used to complement UMLS.

## Background and significance

The analysis of clinical patient data together with experimental data is important in biomedical research and requires principled integration of these data. The ability to integrate real-world data is especially imperative in rare disease research, where disease prevalence is very low and therefore little data are available. The emergence of natural language processing (NLP) tools has made it easier to structure free-text data from the EHR and to map (some) extracted data to the unified medical language system (UMLS), which can then be used to traverse different ontologies.^1^ Ontologies such as the human phenotype ontology (HPO)^2^ and WHO’s international classification of diseases (ICD)^3^ are often used to label patient data. The motivations or use cases for these 2 ontologies are distinct. In the United States, ICD is used in EHRs primarily for reimbursement purposes, while HPO is used for biological characterization in research. These ontologies were developed independently, without coordination to date, and typically have limited interoperability between them. However, with the increasing use and usefulness of electronic health record (EHR) data, which contributes to the “real-world data” characterization of patient populations and diseases, as well as the interest in more detailed characterizations for EHR and cohort databases, the uses of these two ontologies increasingly overlap. This has created an urgent motivation to improve interoperability between ICD and HPO.

While the 2 ontologies do fundamentally cover different scopes, the links between diseases and phenotypes are important, as they allow for the transformation of data from the EHR to the research context where phenotypes are essential in the characterization of diseases in research settings. While there are other ontologies that might be better suited for mapping from diseases in ICD codes to diseases in respective ontologies, like the Monarch Disease Ontology (MONDO), these are not as useful toward disease characterization and clinical diagnostics as some conditions do not have a disease label yet. There is a certain gray area between phenotypic features and diseases. Some diseases can still present themselves in HPO as features of other diseases (eg, diabetes mellitus as a feature of Bardet Biedl syndrome). The extent of overlap is unknown. This exercise quantifies the overlap between these 2 domains of phenotypic features and disease.

To illustrate the lack of interoperability, consider the concept “acute bronchitis,” which is represented in ICD and HPO with different identifiers—J20 in ICD for “acute bronchitis,” and HP:0012388 in HPO. Without a mapping between these 2 symbols to establish that they are conceptually equivalent, and should be interpreted as the same thing, data that rely on only one of the codes cannot be easily cross-analyzed. Although there have been recent developments in NLP tools for ontology mapping purposes, there still lacks a consensus on how to use these tools across research groups to achieve accurate and reusable mappings between ontology terms. For this reason, many researchers rely on the UMLS Metathesaurus, which provides mappings between ontologies derived through expert curation, to attain partial interoperability among the ontologies in UMLS.^4^ The UMLS is widely used for mapping across ontologies with approximately 50% of users using it for that purpose.^5^

A survey we conducted of recent research published in the last 2 years (see Table 1) demonstrates that researchers rely on UMLS as the primary source of mappings between HPO and ICD. Schofield et al developed a method (reported in paper #1 in Table 1) to identify and gather disease-phenotype associations by leveraging the UMLS mappings of diseases represented in ICD to phenotypes from the HPO or mammalian phenotype (MP) ontologies.^6^ In papers #2-#4, the authors used semi-automated mapping tools (specifically MetaMap, CLAMP, and cTakes) to identify HPO terms in free-text descriptions of phenotypes from EHR clinical notes. In the setting up of the open annotation for rare diseases (OARD)—a data resource containing annotations for rare-disease-related phenotypes, described in paper #4—the authors used the NLP tool cTakes to harmonize claim codes, lab procedures, and clinical notes with UMLS concept unique identifiers (CUIs) before traversing to HPO and MONDO ontologies. In paper #5, the authors used existing mappings of ICD to HPO obtained from UMLS and BioPortal, and then a partial-logical mapping strategy that uses the HPO structure in order to obtain better mapping coverage of ICD to HPO terms. In paper #6, the authors applied automated string matching using the bidirectional encoder representations from transformers (BERT) NLP model followed by manual verification of disease name mappings. Paper #7 provides a Phecode-HPO mapping set generated by using mappings in UMLS, the Phecode map of ICD codes to Phecodes, and tool-generated mappings using different approaches. From this short survey, it is evident that recent research heavily depends on UMLS as the main source of mappings, highlighting the need to quantify the biases and implications of these mappings.

**Table 1.** Articles published in 2021-2022 that used mappings between ICD and HPO.

#	Title	Mapping reference(s)	Mapping tool(s)	Year
1	Linking common human diseases to their phenotypes; development of a resource for human phenomics^6^	UMLS	None: employed newly introduced methods involving NLP + UMLS	2021
2	Clinical phenotypic spectrum of 4095 individuals with down syndrome from text mining of electronic health records^7^	UMLS	MetaMap	2021
3	Development of a phenotype ontology for autism spectrum disorder by natural language processing on electronic health records^8^	UMLS	CLAMP	2022
4	OARD: open annotations for rare diseases and their phenotypes based on real-world data^9^	UMLS	cTakes	2022
5	Common genetic variation associated with Mendelian disease severity revealed through cryptic phenotype analysis^10^	BioPortal + UMLS	None: employed a partial logical mapping strategy	2022
6	Building a knowledge graph to enable precision medicine^11^	UMLS	BERT	2022
7	Linking rare and common disease vocabularies by mapping between the human phenotype ontology and phecodes^12^	UMLS, PheMap	SORTA, string-matching, and WikiMedMap	2023

Abbreviations: BERT, bidirectional encoder representations from transformers; HPO, human phenotype ontology; ICD, international classification of diseases; UMLS, unified medical language system.

In this paper, we quantify the limitations and biases of using UMLS mappings of ICD to HPO and how they may affect research findings. Specifically, we set out to determine the coverage of mappings between ICD10-CM—the finer-grained variant of ICD10 used in the United States—and HPO, as this indicates how much information in the EHR is “covered” when transiting between EHR coding (ICD) and research-oriented ontologies (such as HPO), and to discuss what that coverage implies for the (potential) integration and analysis of EHR data with experimental data. (From here on out, any mention of “ICD” is meant as a shorthand for “ICD10-CM.”) We quantify how exhaustively the popular ICD classification has been mapped to HPO by analyzing mappings derived from the widely used source of mappings between biomedical ontologies—the UMLS Metathesaurus. The purpose of our work is not to specify new mappings, nor to identify erroneous mappings; rather we provide a synchronic analysis of the mappings that are publicly accessible and currently used to convert from ICD to HPO for such goals as rare disease analysis. We also discuss the implications of those decisions for secondary research and analysis in the context of real-world EHR data from Beth Israel Deaconess Medical Center (BIDMC).

## Methods and materials

### Ontologies analyzed

The HPO ontology logically and systematically describes phenotypic traits in human diseases. Many public disease knowledge databases, such as MedGen^13^ and Orphanet,^14^ as well as consortiums like the undiagnosed disease network (UDN) use HPO as the controlled vocabulary to annotate phenotypes for diseases. Various algorithms and tools also leverage HPO to support phenotype-based differential diagnostics, gene-disease discovery, and genomic diagnostics, among others. For example, the Phen2Gene tool uses a database of weighted and ranked gene lists for every HPO term, and then, given a patient-specific list of HPO terms, the tool calculates a prioritized gene list based on a probabilistic model and outputs gene-disease relationships.^15^

Real-world patient data from hospitals and health insurance claims data, on the other hand, are often stored using codes from WHO’s ICD, which is primarily designed and used as a billing instrument. There is a vast amount of patient data encoded using these ICD annotations, including signs and symptoms, diseases, external causes of injury or diseases, and abnormal findings.^16^ Currently the most widely used version of ICD in healthcare settings throughout the United States is ICD10-CM, which replaces the older ICD9-CM.

### Sources of ontology mappings

As we mentioned, UMLS appears to be the most popular and comprehensive source of mappings. However, there are other potential sources of mappings that researchers can consider when integrating data annotated with different ontologies. BioMappings^17^ provides a community-contributed table of mappings—some verified by humans, while others simply predicted using software tools. At the time of writing, there were zero mappings between ICD and HPO in the BioMappings repository. Ontologies themselves often include mappings between the terms they define and terms in other ontologies or databases (so-called “database cross-references”). The ICD classification itself does not contain any such mappings; however, HPO does. There are 39 mappings between HPO and ICD terms, with 20 of those only present in HPO, and not in UMLS. These “missing” mappings could be due to UMLS not being up-to-date with the latest release of HPO. We also extracted 632 ICD mappings from the MONDO disease ontology, and which have an HPO mapping. We determined that all 632 mappings are already contained in UMLS.

### UMLS as a current mapping source

The UMLS Metathesaurus unifies concepts across ontologies via an assignment of CUIs. A CUI is assigned to each unique collection of terms that are conceptually equivalent. For our study, we used the ICD CUIs and searched for HPO terms with identical CUIs in the May 2022 version of the UMLS tables. More specifically, after downloading the UMLS archive (umls-2022AA-mrconso.zip), we use in our analysis the file MRCONSO.RRF (of 2GB in size) contained therein—this is a table containing all UMLS CUIs and their associated labels, synonyms, and mappings to different ontologies. The table comes without column names; in our analysis, we primarily use column 1 (CUI), column 12 (SAB—abbreviated source ontology), column 13 (CODE—ontology term identifier), and column 15 (STR—string label). For example, the following sample of the UMLS table contains 2 mappings: (1) between ICD10CM code K85 and HPO term HP:0001735 and (2) between K85.9 and HP:0001735, because they share the same CUI. When a CUI has both an ICD code (*I*) and an HPO term (*H*) associated with it, we say that *I* is mapped to *H*.

CUI | SAB | CODE | STR

C0001339 | ICD10CM | K85 | Acute pancreatitis

C0001339 | ICD10CM | K85.9 | Acute pancreatitis, unspecified

C0001339 | HPO | HP:0001735 | Acute pancreatitis

C0001339 | HPO | HP:0001735 | Acute pancreatic inflammation

In 2014, Bodenreider et al analyzed the coverage of disease phenotypes in standard biomedical ontologies to determine which phenotypes have a mapping in UMLS, and to which specific ontology(ies) in UMLS they map to.^18^ A mapping to UMLS was found for 54% of disease phenotypes, with the best coverage by a single ontology being provided by SNOMED CT, which covers 30% of phenotypes in HPO. According to the study, at the time, ICD10-CM provided coverage for only 15% of HPO phenotype terms.^18^

### Mapping coverage of codes with different levels of usage in BIDMC data

Our BIDMC EHR dataset was extracted in February 2022, and is a subset of the data submitted to the 4CE Consortium.^19^ To understand the mapping coverage of codes with different levels of usage, we split the ICD codes into 3 usage groups. Codes that are used across more than 1% of patients are referred to as common codes; Codes that are used in 1%-0.1% of patients are categorized as infrequently used, and codes that are used in less than 0.1% of patients are grouped as rare ICD codes. For each of these groups, we computed the proportion of mapped and unmapped codes as well as the proportion of codes that fell into the “others” category. Codes in the “others” category are those found in the ICD code list in our EHR dataset, but which did not match existing ICD10-CM codes. Throughout our analyses, we stratified the setting of care into 3 groups: intensive care unit (ICU) patients, admitted patients, and outpatients. The rationale for this stratification is that the setting of care might confound the mapping coverage, since codes used in outpatients could be very different from codes used in ICU patients. Furthermore, use cases for different care settings may differ, so it would be informative to characterize mapping coverage across these patient groups. For example, researchers working with ICU data who want to convert their ICD codes to HPO terms might face a different challenge compared to researchers working with outpatients when performing the same task.

### Mappability of the most used ICD codes

To survey the mappability of the most used ICD codes, we retrieved the 10 ICD codes with the highest number of patient counts without an associated HPO term and those with a mapped HPO term. For each of these codes, we extracted the number of times the codes were used (counts) and the number of patients that have been assigned these codes (Patient Counts). For those with a matched HPO term, the matching HPO term and label are also presented.

### Mappability of ICD code categories by top-level ICD categories

We stratified our next analyses by top-level ICD categories to better understand if certain disease groups are better mapped. To further differentiate the codes, we split them into 2 groups: 1 for less-used codes which are used in less than 100 patients (inclusive), and codes that are more commonly used in more than 100 patients.

We then calculate the PatientCoverage for each of these groups of ICD codes. The purpose of calculating PatientCoverage was to quantify the proportion of patients with ICD codes from each ICD category that have HPO mappings. To calculate this, we take the summation of the total number of patients with mapped ICD codes as a proportion of the total number of patients with codes assigned in the ICD category. For example, consider the codes used in <100 patients from the category “Q: Congenital malformations, deformations and chromosomal abnormalities”—there are 309 codes used a total of 3506 times. Of these usage counts, 1260 involve mapped terms and hence the proportion mapped for this ICD category in terms of usage counts is 1260/3506, resulting in a patient coverage proportion of 0.359.

(1) $$\mathrm{Patient}\;\mathrm{Coverage}=\frac{\Sigma \;\mathrm{Patient}\;\mathrm{Counts}\;\mathrm{for}\;\mathrm{matched}\;\mathrm{codes}\;}{\;\Sigma \;\mathrm{Patient}\;\mathrm{Counts}\;\mathrm{for}\;\mathrm{all}\;\mathrm{codes}\;\mathrm{in}\;\mathrm{category}}$$

## Results

### Dictionary level mapping

We first collated the dictionary-level mapping of all ICD codes to HPO, which is composed of all mappings between ICD codes and HPO terms in UMLS. The results from this analysis show that 2.2% of ICD codes are mappable to HPO via the UMLS Metathesaurus (Table 2). Although the proportion mappable is low, the distribution of mapped codes within real-world hospital ICD usage would provide a practical estimate of the consequences of the sparse mapping.

**Table 2.** Mappings between ICD and HPO in UMLS.

Ontology	# Codes	# *Unique* HPO codes mapped to ICD	% HPO codes mapped to ICD	# *Unique* ICD codes mapped to HPO	% ICD mapped to HPO (#unique codes mapped/total ICD codes)
HPO	16 366	1870	11.4%	–	–
ICD10-CM	95 847	–	–	2066	2.2%

ICD10-CM is the coding system used at BIDMC.

Abbreviations: BIDMC, Beth Israel Deaconess Medical Center; HPO, human phenotype ontology; ICD, international classification of diseases; UMLS, unified medical language system.

### Mapping coverage of codes with different levels of usage

**Figure 1.**
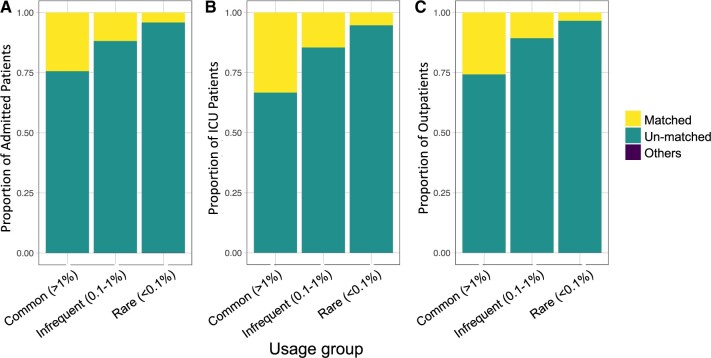
The proportion of ICD/diagnosis codes that are matched to an HPO term, unmatched to any HPO terms or others (do not have a corresponding ICD code in the UMLS dictionary). The proportions were calculated for common codes that are used in >1% of the cohort, infrequently used codes that are attributed to 0.1%-1% of the cohort, and rare codes that are assigned to <0.1% of the patient cohort. These were calculated separately for the (A) admitted patients, (B) ICU patients, and (C) outpatients. HPO, human phenotype ontology; ICD, international classification of diseases; UMLS, unified medical language system.

Therefore, we investigated the mappability of codes across different levels of usage with the categories for commonly used ICD codes (>1%), codes that are infrequently used (0.1%-1%), and codes that are rarely used (<0.1%). For the commonly used codes (>1%), the ICU patients have the highest proportion of mapped codes (33.3%) as compared to the admitted patients and outpatients which have 24.4% and 25.8% of the ICD codes matched to a corresponding matched HPO term, respectively (Figure 1). ICU patients also show a higher proportion mapped for the infrequently used ICD codes as compared to the admitted and outpatient cohorts possibly due to better-defined codes that are used in the ICU (Figure 1). Surprisingly, the outpatient group also does marginally better than the admitted patients for the commonly used codes. Overall, although less than half of the codes used are mappable to HPO via UMLS, it is reassuring that codes that are used more frequently tend to have a higher proportion mapped to HPO.

### Mappability of the most used ICD codes

To survey the mappability of the most used ICD codes, we retrieved the 10 ICD codes with the highest number of patient counts without (Table 3) and with an associated HPO term (Table 4). For example, Table 3 presents some ICD codes that are used in roughly 2000 patients or more, and which do not have a corresponding HPO term, hence representing the greatest loss of information when converting between ICD and HPO terms. Of the codes that are not mapped, there were many (more than 7 out of 10 codes) that were from the ICD category “Z” that covers codes that are “factors influencing health status and contact with health services.” These codes mainly cover encounters with the health system and hence the absence of mappings is expected since the scope of HPO is to describe phenotypes. While we could choose not to interpret these as missing mappings, as we expect them to be missing, we point out that there are some Z codes in ICD that are mapped to HPO—for example, Z67.1 (Type A blood) is mapped to HP:0032370 (Blood group A). So to emulate the process of converting EHR data, we did not remove Z codes from our analysis since we want to characterize the overall mappability of ICD to HPO. Also, this category of codes only accounted for 5.7% of all codes used in admitted patients and does not significantly affect overall mappability.

**Table 3.** The 10 most frequently used codes in admitted patients, ICU patients, and outpatients (not admitted) that do not have a matched HPO term.

Cohort	Counts	Patient counts	CUI	ICD ID	Label
Admitted	15 543	11 671	C5539297	Z20.822	Contact with and (suspected) exposure to COVID-19
	12 544	7184	C0085580	Z87.891	Personal history of nicotine dependence
	11 625	6659	C2911355	Z23	Encounter for immunization
	6283	6219	C0341102	Z37.0	Single live birth
	7006	5750	C2910668	Z20.828	Contact with and (suspected) exposure to other viral communicable diseases
	10 348	5620	C1313895	F41.9	Anxiety NOS
	6721	5155	C0022660	Z11.59	Encounter for screening for other viral diseases
	8740	5042	C2910658	Z00.00	Encounter for adult health check-up NOS
	8073	4467	C0003469	F32.9	Major depressive disorder, single episode, and unspecified
	11 461	4405	C2910579	Z79.01	Long-term (current) use of anticoagulants
ICU	5305	3506	C0085580	Z20.822	Contact with and (suspected) exposure to COVID-19
	6029	2946	C5539297	Z87.891	Personal history of nicotine dependence
	3441	2845	C0022660	D62	Acute posthemorrhagic anemia
	3052	2514	C2911355	J96.01	Acute respiratory failure with hypoxia
	2831	2149	C0154298	Z20.828	Contact with and (suspected) exposure to other viral communicable diseases
	5752	2078	C0341102	Z79.01	Long-term (current) use of anticoagulants
	2512	2010	C2977065	Z66	DNR status
	3910	1960	C2882161	F41.9	Anxiety NOS
	2733	1897	C2910658	Z11.59	Encounter for screening for other viral diseases
	3769	1828	C2911178	Z00.00	Encounter for adult health check-up NOS
Outpatients	1 16 491	78 109	C2910579	Z11.59	Encounter for screening for other viral diseases
	57 453	37 259	C5203670	U07.1	COVID-19
	52 548	33 996	C2910447	Z00.00	Encounter for adult health check-up NOS
	39 471	30 929	C2910668	Z20.822	Contact with and (suspected) exposure to COVID-19
	57 339	29 087	C0085580	Z23	Encounter for immunization
	26 199	20 805	C5539297	Z20.828	Contact with and (suspected) exposure to other viral communicable diseases
	24 703	18 863	C5539296	Z11.52	Encounter for screening for COVID-19
	28 360	13 064	C2910658	F41.9	Anxiety NOS
	16 779	12 076	C0341102	Z00.01	Encounter for general adult medical examination with abnormal findings
	18 014	11 573	C2910448	Z87.891	Personal history of nicotine dependence

Abbreviations: CUI, concept unique identifier; HPO, human phenotype ontology; ICD, international classification of diseases.

**Table 4.** The 10 most frequently used codes in admitted patients, ICU patients, and outpatients (not admitted) that have a matched HPO term.

Cohort	Counts	Patient counts	ICD ID	ICD label	HPO	HPO label
Admitted	36 074	11 273	I10	High blood pressure	HP:0000822	High blood pressure
	23 666	10 802	E78.5	Hyperlipidemia, unspecified	HP:0003077	Hyperlipidemia
	13 486	6791	K21.9	Esophageal reflux NOS	HP:0002020	Gastroesophageal reflux disease
	10 538	6010	N17.9	Acute kidney failure, unspecified	HP:0001919	Acute kidney failure
	14 376	4954	I25.10	Atherosclerotic heart disease NOS	HP:0001677	Coronary atherosclerosis
	10 429	4834	D64.9	Anemia, unspecified	HP:0001903	Anemia
	7986	3908	E66.9	Obesity NOS	HP:0001513	Obesity
	5464	3530	E87.1	Sodium [Na] deficiency	HP:0002902	Hyponatremia
	4358	3386	E87.2	Acidosis	HP:0001941	Acidosis
	9044	3375	E03.9	Hypothyroidism, unspecified	HP:0000821	Hypothyroidism
ICU	10 776	4494	E78.5	Hyperlipidemia, unspecified	HP:0003077	Hyperlipidemia
	14 953	4431	I10	High blood pressure	HP:0000822	High blood pressure
	5596	3045	N17.9	Acute kidney failure, unspecified	HP:0001919	Acute kidney failure
	6049	2655	K21.9	Esophageal reflux NOS	HP:0002020	Gastroesophageal reflux disease
	7573	2422	I25.10	Atherosclerotic heart disease NOS	HP:0001677	Coronary atherosclerosis
	2938	2313	E87.2	Acidosis	HP:0001941	Acidosis
	4597	2032	D64.9	Anemia, unspecified	HP:0001903	Anemia
	3238	1997	E87.1	Sodium [Na] deficiency	HP:0002902	Hyponatremia
	3187	1398	E66.9	Obesity NOS	HP:0001513	Obesity
	1711	1385	I95.9	Hypotension, unspecified	HP:0002615	Hypotension
Outpatients	85 112	23 465	I10	High blood pressure	HP:0000822	High blood pressure
	44 506	18 526	E78.5	Hyperlipidemia, unspecified	HP:0003077	Hyperlipidemia
	30 216	14 182	K21.9	Esophageal reflux NOS	HP:0002020	Gastroesophageal reflux disease
	15 559	9204	R05	Cough	HP:0012735	Coughing
	19 115	8927	E66.9	Obesity NOS	HP:0001513	Obesity
	13 624	7858	R07.9	Chest pain, unspecified	HP:0100749	Chest pain
	13 525	7396	R53.83	Fatigue NOS	HP:0012378	Fatigue
	18 379	6982	G47.33	Obstructive sleep apnea (adult) (pediatric)	HP:0002870	Obstructive sleep apnea
	9565	6947	R51.9	Headache, unspecified	HP:0002315	Headache
	13 015	6575	R06.02	Shortness of breath	HP:0002094	Dyspnea

Abbreviations: HPO, human phenotype ontology; ICD, international classification of diseases.

The other codes that are poorly mapped range from ones like “anxiety NOS” (where NOS stands for “not otherwise specified”) and “major depressive disorder, single episode, unspecified,” “acute posthemorrhagic anemia,” to “acute respiratory failure with hypoxia” which are common diseases with multiple possible causes. Of these unmapped codes, for example, “acute respiratory failure with hypoxia,” a manual lookup on the HPO website using keywords “respiratory failure” yielded a close match “Respiratory failure HP:0002878” which could potentially be mapped to the ICD code and hence better capturing the biology of the 2514 patients associated with the ICD code “J96.01 acute respiratory failure with hypoxia.” This suggests that such trivial manual improvements to mapping bring about a very favorable effort to pay-off ratio in bringing about a much-improved patient representation. If capturing the overall population diagnosis is the goal, then these codes represent the lowest hanging fruits for the highest improvement in patient diagnosis representation. It also points the way to effective use of machine learning techniques for such mappings.

The most frequently used codes which are mappable to HPO terms via UMLS represent the common diagnoses that are most well represented. When studying common diagnoses, one can reasonably assume that the mapping between ICD and HPO would sufficiently capture the prevalence of those diagnoses in the EHR data. However, it should be cautioned that studies looking into diagnoses across diseases would have an overrepresentation of diagnoses in Table 4 since they are better mapped than those in Table 3.

### Mappability of ICD code categories by top-level ICD categories

**Figure 2.**
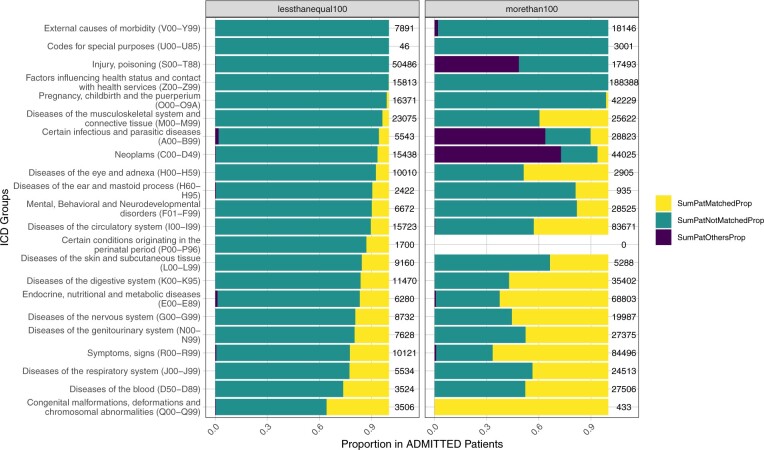
Patient coverage of mapped and unmapped terms across different ICD categories. The left column specifies codes that are used in less than 100 patients, while the right column specifies codes that are used in more than 100 patients in our dataset. The figure depicts in yellow the frequency of usage of ICD codes that have an HPO mapping, in green the usage of ICD codes that do not have an HPO mapping, and in purple the codes in the “others” category which are in the ICD code list of our EHR dataset, but which did not match existing ICD10-CM codes. The numbers on the right of each column specify the number of times that codes from that category are used. HPO, human phenotype ontology; ICD, international classification of diseases.

As the top-level ICD categories represent different major disease groups (eg, category I is for diseases of the circulatory system, category J is for diseases of the respiratory system), the next analysis is stratified by these categories to better understand any biases in the mapping of these disease groups (Figure 2). The outcome from this analysis shows that while codes that are used in less than 100 patients generally have a lower proportion mapped of less than 40% across all ICD categories, codes that are used in >100 patients have a much higher representation in the proportion of patients with a mapped code although the patient coverage does vary substantially depending on the ICD category (Figure 2). We conducted the same analysis of outpatient and ICU patient data, and the results are similar and in line with those of admitted patients that we just discussed (results not shown).

## Discussion

To leverage the vast amount of real-world patient data with the tools developed to work with ontologies, an accurate mapping between the different systems used for annotating these datasets is necessary. In this paper, we have analyzed how well aligned the ICD is with the HPO. The rationale for moving from ICD codes to HPO terms is that, for example, genomics-based diagnostic pipelines rely on standardized phenotype and disease ontology terms as the input, such as terms from HPO. Only with a mapping from ICD-coded patient data to HPO can researchers accurately integrate and cross-analyze data originating from EHRs together with public research data beyond their study. For example, tools like PheRS, which measures the similarity between an individual’s diagnosis codes and phenotypic features of known genetic disorders, also require mappings that link ICD codes to HPO terms, which most EHRs do not contain.^20^ In such scenarios, unless the researcher is an expert in ontology, they will most likely turn to resources like the UMLS tables for these mappings; hence, determining the coverage of ICD10-CM to HPO mappings with the UMLS table is imperative.

We set out to analyze the extent to which ICD codes—either specified in ICD10-CM (ie, our “dictionary level” analysis) or used in our EHR data—were mapped to the widely used phenotype ontology HPO. In our analysis of the mapping coverage, we found that the proportion of terms mapped between ICD and HPO are low both at the dictionary level and in real-world EHR data. That said, the proportion of mapped terms in our EHR data is higher than that found at the dictionary level. However, to enable the integration and cross-analysis of clinical and experimental data at scale, we argue that there is an urgent need to improve mappability across these ontologies, and to make such mappings available to the research community using such vehicles as the UMLS or other appropriate mechanisms. We also found that certain terms have much higher leverage in improving patient coverage and should be looked into first. For example, such ICD codes as those related to COVID-19 infection or other viral diseases, nicotine dependence, and anxiety are each used to annotate over 10 000 patients in our dataset. It should also be cautioned that the proportion of ICD codes mapped to HPO is especially low for rarer conditions (eg, ICD codes from the neoplasm category are considerably less mapped than codes from the nervous system category) and could implicate biases if the data are used as is after conversion.

Our study indicates that the coverage of ICD to HPO mappings has not increased over the years; in fact, the opposite—coverage decreased. This likely occurred because ICD and HPO grew over time (ie ontology terms were added) while the mapping set between the 2 artifacts has seemingly not kept up with their evolution. In the last analysis of mapping coverage reported in the literature in 2014, 15% of HPO codes were mapped to ICD10-CM, whereas currently our analysis indicates that the percentage of HPO terms mapped to ICD10-CM decreased to only 11% in the 2022 version of the UMLS Metathesaurus. The UMLS website provides a similar statistic that roughly confirms our empirical finding, albeit UMLS’s statistics are likely more up-to-date—according to the UMLS website, in November 2023 a total of 10.2% of HPO terms are mapped to ICD10-CM.^21^

It should be noted that there is a mismatch in granularity between the 2 artifacts under analysis, with ICD10-CM being a more coarse-grained representation than HPO. So understandably the proportion of exact mappings between terms is rather low. In certain circumstances, one could consider non-exact mappings between terms in these ontologies—for example, broad and narrow mappings between terms that take into account the hierarchical structures of ICD and HPO—which can still be useful for large data analytics or other data integration purposes. However, to our knowledge, there are no off-the-shelf tools that can identify such broad or narrow mappings. Furthermore, currently, there are no benchmarks or means by which we could ascertain the validity of such mappings, given the lack of publicly available broad/narrow mapping sets between ontologies that researchers could use. In future work, we intend to broaden our analysis to consider other mapping resources (eg, broad/narrow mappings, concept sets) and to determine the extent to which differences in granularity between ICD and HPO can be bridged using such resources.

The scientific value of having a go-to resource for expert-verified mappings (such as UMLS) must not be underestimated. Such a resource allows for turnkey conversion between metadata that describe scientific data using different ontologies or coding mechanisms. It is essential to have appropriate tooling to allow researchers to easily leverage these mappings for their purposes, without having to go through literature, broken links, tables with various formats, and so on. UMLS is a good starting source for mappings, but clearly, there is a need for more comprehensive mapping between ontologies that can complement the current ones. When ontology mappings for entities of interest are not available in a trusted source like UMLS, researchers tend to rely on NLP tools—some of which are based on modern, neural network-based language models—to generate mappings between their codes or ontology terms of interest. Language models are statistical methods that learn the probability of word occurrences based on examples of text in order to make predictions involving those words, and they are used to power NLP applications. For example, the popular language model BERT generates numeric (vector) representations of strings, which can then be compared in vector space using measures such as cosine distance. A comparison between 2 such entities will yield a distance score that researchers can use to determine how similar the 2 entities are in their meaning. In a similar vein, large language models (LLMs) can be helpful in generating tentative mappings between ontologies. While the effectiveness of such approaches has yet to be evaluated, their outputs will certainly require review by qualified human curators and should not be used out-of-the-box. The reproducibility of mappings produced using generative artificial intelligence (AI) models also needs to be considered. While we observed in our survey that such automated approaches are often used in practice, the mappings that researchers generate using these tools and use in their studies are rarely made publicly available—and so other scientists cannot reuse them, or even begin to try to replicate the original experiments that were done using those mappings. Even when researchers share their mappings, they are rarely (if ever) contributed to some centralized repository or registry of mappings that the wider community can leverage, such as UMLS.

## Conclusion

The low number of direct mappings between ICD and HPO implies these are unlikely to be sufficient for fine-grained phenotyping using EHR data. Our results also show that ICD terms with lower usage are less well mapped; hence, the UMLS mappings are less likely to cover any existing ICD codes that might be a component of a rare disease. At best, the current state of mapping resources allows researchers to analyze relatively small cohorts whose diagnoses fall under the popular ICD categories that have been mapped.

## Data Availability

To replicate this analysis it is necessary to have (1) the UMLS Metathesaurus (version 2022AA) and (2) the BIDMC patient dataset: UMLS is freely available upon registering for a UMLS Terminology Services (UTS) account (https://uts.nlm.nih.gov/uts/signup-login). The UMLS Metathesaurus version 2022AA that we used in our study can be obtained from: https://tinyurl.com/m7976xcz. The BIDMC dataset contains confidential patient electronic health data and cannot be shared outside of the institution. Interested parties may contact authors for potential collaboration or contact BIDMC’s Institutional Review Board (IRB) to request access to the data through: https://www.bidmc.org/research/research-and-academic-affairs/clinical-research-at-bidmc/committee-on-clinical-investigation-irb.

## Code availability

The code to replicate the analysis (tables, figures, etc) is available on GitHub at: https://github.com/hms-dbmi/ICDtoHPOmappings.
